# The Need for and Importance of Thorough and Comprehensive Studies on the Molecular Mechanisms of Action of Animal Toxins, Venoms, and Antivenoms

**DOI:** 10.3390/ijms262211007

**Published:** 2025-11-13

**Authors:** R. Manjunatha Kini, Yuri N. Utkin

**Affiliations:** 1 Department of Biological Sciences, National University of Singapore, Singapore 117558, Singapore; dbskinim@nus.edu.sg; 2 Laboratory of Molecular Toxinology, Shemyakin-Ovchinnikov Institute of Bioorganic Chemistry, 117997 Moscow, Russia

Many species of animals are commonly referred to as venomous, as they can produce special secretions known as venoms for defense and/or attack/hunting. Of these, snakes, scorpions, spiders, and stinging insects are the most well-known. The effect of their venoms, which are complex mixtures of various compounds, mainly peptides and proteins, usually leads to a disturbance in the normal physiological functions of the body and, in extreme cases, ends with the death of the victim. It should be noted that the term “venom” is still a subject of debate, particularly concerning its relation to the term “poison”. Broadly, venoms are actively injected/introduced into the prey/predator/victim, while poison passively enters the organism via ingestion (Figure 1). There are several publications considering this subject in detail [1,2,3]. However, in many languages, these concepts do not differ. In quite recent publications, the expansion of the concept of venom has been proposed, for example, to consider plants as victims [4,5]. In this Special Issue “Molecular Mechanisms of Animal Toxins, Venoms and Antivenoms 2.0”, we considered venoms in a narrower sense, as secretions produced by animals and used to subdue prey.

Although toxins in venoms are evolutionarily assembled to effectively and quickly disrupt the normal physiological functions of organisms, in some cases, toxins can have a beneficial effect. A detailed study of venoms is a promising area of research, since toxins can serve as tools for studying the molecular mechanisms of the functioning of various body systems or as a basis for the design of new drugs [6,7]. Intensive research is currently underway, and numerous attempts are being made to introduce drugs derived from animal venoms into clinical practice. However, to make the application of these substances possible, extensive preclinical trials are required, and some applications also require clinical trials to confirm their molecular targets, mechanism of action, effective doses, and potential side effects, as well as other fundamental parameters. Nevertheless, every year, advances in the study of the structure and mechanisms of the action of toxins on specific targets in the body bring us closer to the creation of a new generation of drugs for the treatment of cardiovascular diseases, pain, cancer, and other socially significant diseases.

One of the main advantages of venoms as sources of new biologically active compounds is their structural and functional diversity (e.g., [8,9]). Toxins are extremely diverse and, in the process of evolution, have acquired the ability to affect numerous vital systems of prey organisms. Many toxins evolved from normal physiological proteins, and various evolutionary mechanisms, including gene duplication, so far play an important role in the expansion of toxin families in venom. This diversity is becoming increasingly successfully deciphered with the development of new methods for analyzing the structure and biological activity of complex natural compounds. Thus, the analysis of the protein composition of venom, proteomics, and the analysis of the composition of mRNAs encoding toxins in the venom gland, transcriptomics, in simultaneous application to the analysis of venoms, gave rise to venomics [10,11,12]. From quite recent developments, the application of micro- or even nanofractionation and biosensors for venom analysis should be mentioned, for example, the methods developed by the Dutch research group (Prof. Kool) [13,14]. In this Special Issue, they presented the results of the analysis of interaction of snake venom neurotoxins with acetylcholine-binding protein (AChBP), using nanofractionation analytics and a post-column plate reader-based fluorescence-enhancement ligand displacement bioassay [15]. The idea was to test the AChBP as a neurotoxin binder for the alternative treatment of elapid snakebites. However, while AChBP was able to effectively bind long-chain neurotoxins, it showed low or no binding affinity towards short-chain neurotoxins, thus demonstrating limited toxin-neutralizing potency.

Animal venoms produce their effects through precise molecular interactions, utilizing their toxins to disrupt host physiological systems including nervous, cardiovascular, and immune systems. Toxins act on specific molecular targets, resulting in neurotoxicity, inflammation, blood coagulation issues, and tissue degradation. The specific mechanisms vary greatly, depending on the venomous animal. Obviously, the nervous system is the main target for animal venoms, since disruption of its functions leads to rapid and effective immobilization or death of the victim [16]. Neurotoxins target ion channels, receptors, and enzymes of the nervous system, interfering with nerve impulse transmission [17]. The neurotoxin targets are quite complex molecules, which often comprise several subunits playing different roles in toxin–target interaction. This Special Issue includes an article in which the possible role of the β subunit of voltage-gated sodium channels in resistance to the well-known neurotoxin tetrodotoxin is discussed [18]. The authors hypothesize that, in addition to the main α subunit, β subunits may play an important role in the evolution of TTX resistance.

An important target for animal venoms is also the cardiovascular system. In this system, toxins can affect blood coagulation, blood vessels, and heart function, as well as trigger inflammatory responses by affecting endothelial cells [19,20]. However, it should be noted that not all the cardiovascular effects of animal toxins are negative. Several animal toxin-based medications have been introduced into practice, with perhaps more than half of them affecting the cardiovascular system [21,22]. Here, the antihypertensive captopril should be mentioned, as it was the first drug designed based on an animal venom component, bradykinin potentiating peptide from the jararaca (*Bothrops jararaca*) snake [23]. Blood clotting disorders are a serious medical condition that, if left untreated, can lead to serious illnesses such as stroke or myocardial infarction. Animal venoms contain toxins that modulate the mammalian coagulation and fibrinolytic systems, and some of these toxins are used in diagnostic assays and as therapeutic drugs [24]. However, the need for new drugs that selectively affect certain stages of blood clotting is still high. In this Special Issue, a novel cystine-knot peptide from spider *Macrothele* sp. venom was identified [25], which has an inhibitory effect on blood clot formation, increasing the activity of the activated protein. This finding may lead to the design of a new therapeutic agent targeting intravascular coagulation in arteries and veins.

The immune system is not considered a target for animal venoms or toxins as often as the nervous or cardiovascular systems. However, there are several animal toxins that affect the immune system. Components of both the adaptive and innate immune systems can be stimulated or suppressed [26]. The best-known toxin affecting the immune system is probably cobra venom factor, which can deplete components of the immune system [27]. Some venom produces an acute inflammatory response, and to prevent negative consequences and stop it, it is necessary to understand the molecular mechanism of its origin. In this Special Issue, one such study focuses on the immunopathology associated with *Bothrops lanceolatus* snake envenomation [28]. The results obtained showed that venom phospholipase A2 triggered several immunopathological events and may contribute to the thrombotic disorders present in the envenomed individuals.

The above discussion shows that animal toxins affect various vital body systems and multiple biological targets. It is believed that most toxins exert influence on their biological target with high efficiency and selectivity, making them good molecular tools for fundamental research and an ideal basis for drug development. However, this is not so definitely and recent data show that some toxins, previously thought to be highly selective for a particular biological target, are capable of interacting with other ones. For example, the α-neurotoxins of snake venom were previously considered to be highly selective inhibitors of nicotinic acetylcholine receptors. However, recently, it was shown that α-bungarotoxin and α-cobratoxin, widely used as selective markers of nicotinic acetylcholine receptors of the muscle and α7 types, are capable of interacting with ionotropic GABA A receptors [29,30]. There is also evidence that α-cobratoxin may activate the M4 muscarinic acetylcholine receptor [31]. However, the two to three orders of higher magnitude in affinity and selectivity windows, along with biological distribution and compartmentalization issues, contribute to the specific pharmacology of these toxins. Data on new biological targets for other animal toxins are also emerging. Thus, phospholipases A2 have been shown to inhibit nicotinic acetylcholine receptors [32,33]. The biological targets for animal toxins lacking enzymatic activity are predominantly proteins. The exceptions are perhaps membrane-active peptides and cytotoxins that interact with lipid membranes. Cytotoxins have also been shown to interact with certain carbohydrates [34]. Work published in this Special Issue shows for the first time that nucleic acids can act as possible biological targets for non-enzymatic toxins [35]. It was found that three-finger toxins, forming one of the largest families of snake venom toxins, are able to bind natural DNA and RNA. Therefore, some biological effects, such as cytotoxin-induced apoptosis in cancer cell lines, may be mediated by the interaction of three-finger cytotoxins with nucleic acids. Which is more productive in terms of the toxin’s destructive capacity: being highly selective or widely effective? There is no definitive answer; everything depends on the specific animal species, its habitat, living conditions, diet, etc.

In conclusion, the papers published in this Special Issue demonstrate the need for and importance of further comprehensive and thorough study of the molecular mechanisms of action of animal venoms and toxins. The results obtained can serve as a basis for developing effective treatments for bites and stings from venomous animals, as well as for the design of new drugs.

## Figures and Tables

**Figure 1:**
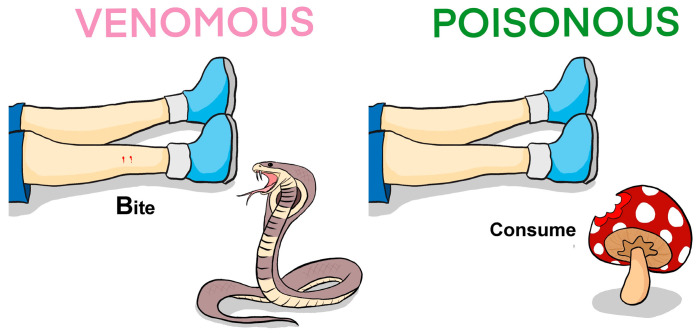
Comparison of venomous and poisonous organisms. A venomous organism ‘actively’ injects venom into the prey/victim, while the victim consumes the poisonous organism to ingest the poison. Thus, venomous animals include snakes, scorpions, spiders, bees, ants, etc., whereas poisonous organisms include mushrooms, amphibians, fishes, plants, etc. Venom and poison contain toxins that interfere with various physiological functions of the victim.
